# Colonoscopy Quality Assurance and Maintenance of Competency Among Pediatric Gastroenterology Staff Members: A Canadian Center Experience

**DOI:** 10.7759/cureus.26126

**Published:** 2022-06-20

**Authors:** Meshari Alaifan, Collin Barker

**Affiliations:** 1 Pediatric Gastroenterology, Faculty of Medicine, King Abdulaziz University, Jeddah, SAU; 2 Pediatric Gastroenterology, BC (British Columbia) Children Hospital, Vancouver, CAN

**Keywords:** (ti) terminal ileum, (ibd) inflammatory bowel disease, (asge) american society of gastrointestinal endoscopy, (crc) colorectal carcinoma, (tii) terminal ileum intubation

## Abstract

Introduction

Colonoscopy quality indicators and maintenance of competency skills are relatively well established in the adult literature as compared to the pediatric gastroenterology. One of the suggested quality assurance measures is cecal intubation rate, which is suggested to be >90% in all colonoscopies as per American Society of Gastrointestinal Endoscopy (ASGE) guidelines. Terminal ileum (TI) intubations are essentially required for diagnostic reasons in pediatric colonoscopies as compared to the screening reasons in adults. Maintenance of competency in pediatric colonoscopies has been described in the literature but in smaller studies contrary to the adult ones. The aims of this study are to compare our center’s individual and group cecal intubation rates and compare it with the published literature, assess the group’s terminal ileal intubation rates in comparison with the published literature, assess the most common reasons for failure to intubate the cecum and/or terminal ileum, and to assess whether the presence of a trainee affects the intubation rates and the duration of the procedure.

Methods

A retrospective chart review was performed on all pediatric patients (0-18 years). Colonoscopies performed over a two-year period at our single center were included in the study. Patients scheduled for sigmoidoscopy and with altered anatomy of their colon were excluded from the study. The endoscopy and pathology reports were reviewed to ascertain whether the cecum and TI were reached. Quality of bowel preparation and any other stated reasons for incompletion were obtained. Clinical charts were reviewed to obtain indication for colonoscopy. Skin-to-skin time, which is the time from starting to the finishing of the procedure, was recorded for each procedure.

Results

A total of 391 colonoscopies were performed during the two-year study period by six gastroenterologists. The number of colonoscopies per staff ranged from 57 to 89 procedures. The overall cecal intubation rate was observed to be 98.5% (range: 95.9%-98.9%). TI intubation rate was lower at a rate of 83.1% (range: 63.3%-92.1%). The main stated reason for the inability to attain cecum/TI was technical difficulty and poor bowel prep. Daytime colonoscopies were shorter (39.5 minutes vs 50.3 minutes) compared to after-hours ones and had a higher TI intubation rate (84.5% vs 62.5%). No complications were encountered in the procedures.

Conclusion

Despite relatively low volumes, cecal intubation rates are very high, exceeding suggested standards. TI intubation rates were low, and there was noted to be a high degree of variability. However, multicentric collaborative evaluations are required over a longer period of time to establish relevant parameters for quality assurance and competency in pediatric endoscopy.

## Introduction

Quality standards are being developed for patient safety and quality care throughout the world. Quality indicators and maintenance of competency skills for colonoscopy are relatively well established in the adult gastroenterology literature. One of the suggested quality measures is cecal intubation rate, which is a paramount requirement for the complete assessment of colonic polyps. It is suggested to be >90% in all the colonoscopies as per the guidelines of the American Society of Gastrointestinal Endoscopy (ASGE) [1]. In pediatric endoscopy, quality measures are still in the developing stage and are not as well defined as in adults [2].

Since the majority of adult colonoscopies are performed for colorectal carcinoma screening, the end point assessed is cecal intubation success rate. However, the indications for pediatric colonoscopies are different. These include investigation for inflammatory bowel disease (IBD), bleeding per rectum, and polyp screening [2]. Additional reported indications include abdominal pain and diarrhea [3].

All of these indications generally require the assessment of the terminal ileum in addition to the entire colon. The Porto criteria for the work-up of potential IBD patients suggest terminal ileum intubation [4]. Thus, terminal ileum intubation (TII) rates may serve as a better indicator of competency given that it is required in a vast number of pediatric cases. The intubation of the terminal ileum is a learned skill which is both difficult and time-consuming.

Maintenance of competency in colonoscopy skills has not been described explicitly in pediatric gastroenterology. A centrally conducted pediatric database study (Pediatric Endoscopy Database System-Clinical Outcomes Research Initiative (PEDS-CORI)) has overviewed the overall intubation rates, but the actual competence and number of procedures performed individually have not been assessed [5]. Few studies have addressed the pediatric competency in pediatric colonoscopies with variable outcomes and results [5-8]. A published paper from Great Britain has defined 120 as the minimum acceptable number of colonoscopies per year to maintain competence in adults [9]. There are many other practice guidelines that suggest a minimum number of colonoscopies per year in order to maintain competency and improve colorectal cancer identification or adenoma detection rate [10]. However, in case of pediatric endoscopy, no such data are available in the literature yet. An effort has been done for assessing the competency of trainees in endoscopy skills in fellowship programs but not the maintenance of skills of attending physicians/consultants [11].

Objectives

The aims of this study are to compare our center’s individual and group cecal intubation rates and compare it with the published literature, assess the group’s terminal ileal intubation rates in comparison with the published literature, assess the most common reasons for failure to intubate the cecum and/or terminal ileum, and assess whether the presence of a trainee affects the intubation rates and the duration of the procedure.

## Materials and methods

A retrospective chart review was done for all pediatric patients (age: 0-18 years) at British Columbia Children’s Hospital. All the colonoscopies performed at our center during a two-year period were included. Patients scheduled for sigmoidoscopy and with altered anatomy of their colon were excluded from the study. The endoscopy and pathology reports were reviewed to ascertain whether the cecum and terminal ileum (TI) were reached. The data were collected from four sources: hospital electronic charts (Powerchart, Cerner, USA), the gastrointestinal division clinical charts, the endoscopy electronic data reports, and the operating room records. The demographic details of the patients included age, gender, and ethnicity. Procedure data included indication for procedure, cecal and terminal ileum intubation success, duration of procedure, reasons for incomplete cecal and terminal ileum intubation, presence of a fellow, quality of colonic cleanout, and whether the procedure was performed in a regularly scheduled endoscopy time or after-hours. The fellow’s role was not clearly stated as the primary person performing the procedure or assisting the staff member. After-hours were defined as after 5 pm, during weekends, or statutory holidays. Skin-to-skin time and procedural time were also obtained from operating room records. The staff gastroenterologists were assigned numbers to assist in blinding. All patient procedures were assigned unique identification numbers for blinding purpose. The study was submitted for ethical approval; however, the committee waived the need for approval, given the nature of the study.

## Results

Six gastroenterologists performed a total of 391 colonoscopies during the two-year period. The number of colonoscopies per staff ranged from 49 to 89 procedures during that period. The total number of patients was 380, of which 232 (61%) were males. Caucasian ethnicity was the highest (70.6%) followed by South Asian (14.6%). The overall cecal intubation rate was 98.5% (ranging from 95.9% to 98.9%), and TI intubation rate was 83.1% (ranging from 63.3% to 92.1%) (Table 1).

**Table 1:** Cecal and terminal ileal intubation rates per staff member and overall for the group.

Staff	Total Scopes (N)	Incomplete Cecal Intubation (N)	Success Rate (%)	Incomplete TI Intubation (N)	Success Rate (%)
1	89	1	98.9%	7	92.1%
2	58	1	96.6%	8	86.2%
3	67	1	98.5%	11	83.5%
4	49	1	98%	18	63.3%
5	57	1	98.2%	13	77.2%
6	71	1	98.6%	9	87.3%
Total	391	6	98.5%	66	83.1%

A total of six colonoscopies had incomplete cecal intubation, with an overall success rate of 98.5%. The incomplete cecal intubations were also considered incomplete TI intubation by default. Technical difficulty was the most common reason for not intubating the cecum or the TI and was defined as the inability to intubate without stating the actual cause of the difficulty. The main stated reason for the inability to enter cecum was technical difficulty (two), poor preparation (two), and colonic stricture (two). The overall TI intubation rate was 325/391 = 83.1%. The main reasons for not intubating TI were technical difficulty (30/391 = 7.7%), poor preparation (16/391 = 4.1%), unable to visualize (3/391 = 0.8%), stricture (3/391 = 0.8%), inflamed ileocecal valve (5/66 = 1.3%), and was not attempted or given indication was not clear (9/66 = 2.3%). Based on the age, patients who are equal to or less than five years of age have the lowest rate of TI intubation rate (72.3%) compared with the rest of the age groups (Table 2).

**Table 2:** Terminal ileum intubation rates based on age group.

Age Group	No. of Scopes	TI Intubation Rate
≤5	47	72.3%
6-10	72	87.5%
11-15	204	83.3%
16-18	68	85.3%

**Figure 1:**
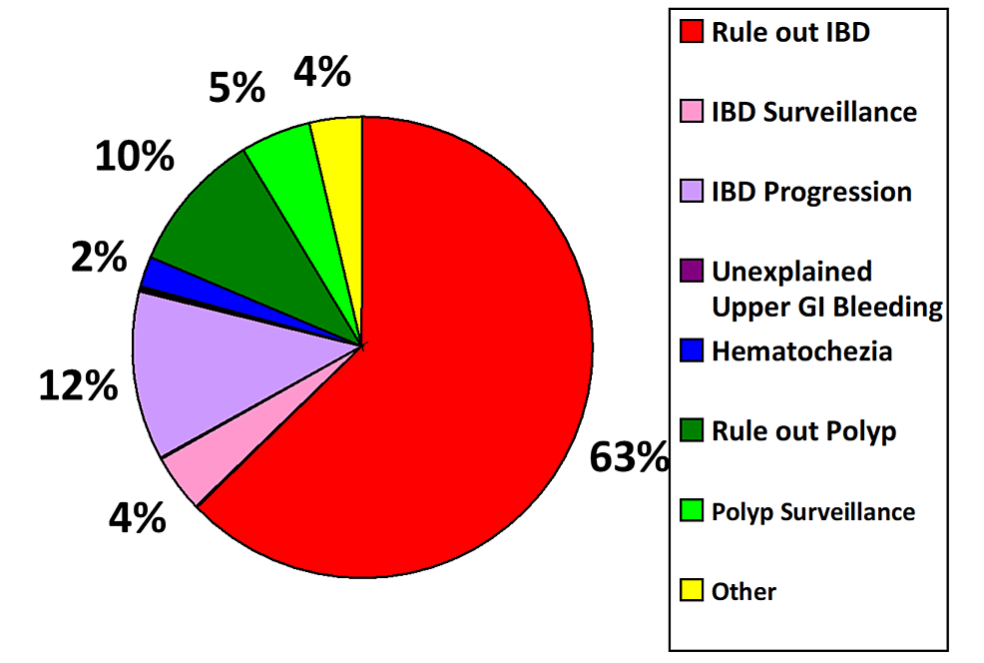
Indications for colonoscopies. IBD: inflammatory bowel disease.

Main indications for colonoscopies were evaluation for IBD (63%), progression of IBD (12%), evaluation for polyp (10%), polyp surveillance (5%), IBD surveillance (4%), gastrointestinal bleeding (2%), and others (4%) (Figure 1).

Trainees/fellows were present in 277/391 (70.8%) colonoscopies. TI intubation in the presence of trainees/fellows was 326/391 = 83.4%. The extent of the fellow’s role during each procedure was not clearly described in the charting; as a result, it is difficult to interpret the effect of their presence, in addition to their varying level of training. They either performed independently or needed assistance. The mean time was around 42 minutes. Overall mean time needed to complete a colonoscopy was 40.1 minutes per procedure ranging from a mean of 32 minutes to 44.9 minutes per endoscopist (Table 3).

**Table 3:** Procedural time per staff member in minutes.

Staff	Time Total (min)	Mean Time (min)
1	4,089	45.4
2	1,926	33.2
3	2,332	34.8
4	2,356	48
5	2,480	43.5
6	2,840	39.5

**Figure 2:**
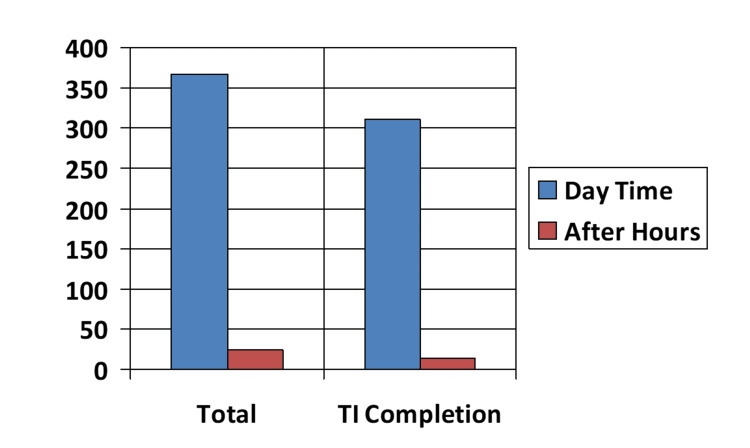
Procedure occurrence, daytime or after-hours. TI: terminal ileum.

Most of the colonoscopies, that is, 367/391 (93.9%), were performed in the regularly scheduled endoscopy time. The regularly scheduled endoscopy colonoscopies were shorter in duration, that is, 39.5 minutes, compared to the after-hours procedures, which was about 50.3 minutes, and had a higher TI intubation rate of around 84.5% as compared to 62.5% for the after-hours procedures (Figure 2). 

The number of after-hours procedure varied among endoscopists from two to seven procedures per endoscopist ranging from 3.4% to 14.3% of their total procedures. Endoscopist four has the longest mean time for a procedure of 48 minutes but has the highest number of procedures done after-hours (14.3%). No complications were encountered in any of the procedures reviewed.

## Discussion

The mean cecal intubation rate observed during the procedures was 98.5% (ranging from 95.9% to 98.9%). Our center’s cecal intubation rates follow the adult recommendations for quality assurance [1,12], despite the lower volumes of pediatric patients compared to adults. The overall TI intubation rate was 83.1%, which was well above the required TI intubation rate required for competency during training [13]. However, it is not as high as reported in the United Kingdom [14,15]. So, it becomes slightly difficult to establish a standard number of procedures to maintain the competency of this skill in practice compared with that of adult literature [8,9]. Despite the lower number of procedures, the overall TI intubation rate at this center was acceptable in the context of historical comparisons for non-United Kingdom reports. The recent pediatric studies mentioned variable percentages of 69%-92.4% [5-8]. The TI intubation rates were found to be quite variable among individuals. Of note, staff number four had the lowest number of carried out procedures (n=49) and also had a lower TI intubation rate (63.3%) (Table 1). On the other hand, staff number one carried out the highest number of colonoscopies (n=89), with the highest percentage of complete TI intubation (92.1%). Overall, a trend was seen with respect to the higher the numbers of procedures performed, the higher the chances of success. This trend was also observed in many other adult studies [8]. 

The main indication for colonoscopy at our center was related to IBD, either for diagnosis or surveillance. This signifies the importance of TI intubation as an essential skill to master, and a great effort should be exercised to maintain this skill further. The majority of colonoscopies in adults are performed for colorectal cancer (CRC) screening, making cecal intubation a justified end point. Other indications, such as ruling out polyps, do not necessitate the need for TI intubation even if the polyp is found. Therefore, the need for TI intubation for every procedure is questionable in adult colonoscopy [16,17].

**Figure 3:**
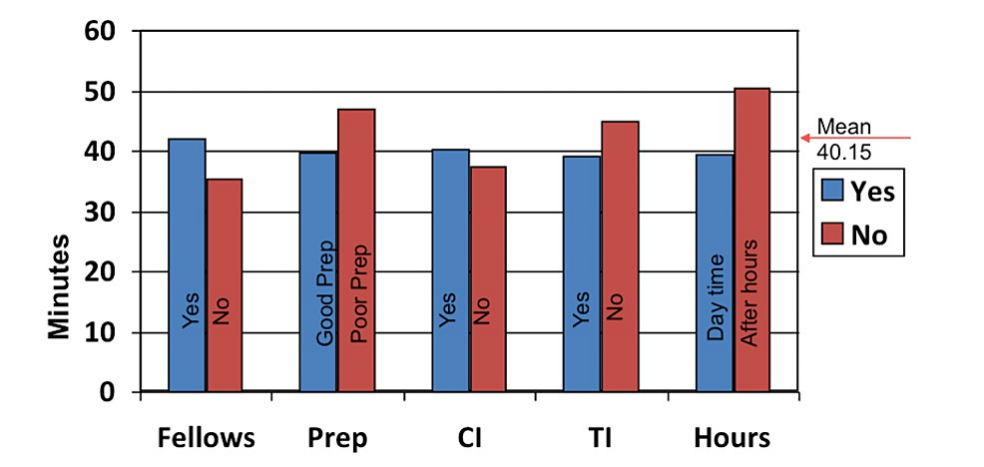
Various factors potentially affecting the duration of colonoscopy. Prep: bowel prep, CI: cecal intubation, TI: terminal ileum.

The incidence of small intestine polyps in hamartomatous-type polyposis syndromes, such as Peutz-Jegher’s syndrome and juvenile polyposis syndrome, is variable [18,19]. Hence, we propose that TI intubation be recommended for colonoscopy procedures with polyps as an indication. It can also serve as both diagnostic and educational experience for the fellows/trainees performing the procedure in the centers with training programs. The mean time for each procedure was 40.15 minutes (ranging from 33.2 to 48 minutes). There were no clear factors per individual that could explain this variation. Even the years of experience was not a contributing factor. Figure 3 shows various reasons affecting procedural timing. Poorly prepped colon and procedures done after-hours took longer times, which were around 45 and 47 minutes, respectively.

After-hours procedures take longer, possibly due to the urgent nature of the procedure, which can be more complicated, especially with IBD patients. The lack of pressure to complete the procedure in time when compared with the procedures scheduled in regular daytime hours is another potential factor. In addition, there is often a lack of regular nursing and supporting staff after-hours which is likely to contribute to longer procedure times after-hours. It is quite interesting to notice the increase in TI intubation rates in pediatric colonoscopies in the recently published data, suggesting increasing awareness among pediatric gastroenterologists about the need for it (Table 4) [20-23].

**Table 4:** Reported terminal ileal intubation rates at various centers over time.

Year	TI Intubation Rate (%)	Author
1994-1996	21.5%	Batres et al. [20]
1999-2000	55.6%	Batres et al. [20]
2001-2010	52%	Kawada et al. [21]
2000-2011	69%	Thakkar et al. [5]
2004-2009	72%	de Bie et al. [22]
2009-2011	89%	Thomson and Sharma [15]
2012-2014	98%	Thomson and Sharma [15]
2012-2015	85%-98%	Saha et al. [14]
2015	91%	Pasquarella et al. [6]
2011-2015	92.4%	Singh et al. [7]
2010-2015	77%	Lee et al. [8]
2015-2016	100%	Thomson et al. [23]

Limitations

This was a retrospective study carried out at a single academic center. The study started off as a quality assurance project with a limited scope to assess. The study time was limited to duration of two years, and the number of procedures per staff was not very high. Therefore, extrapolation to other centers with different patient volumes may not be valid. A prospective, multicenter study would be the next step to assess the physician's endoscopic competency for basic diagnostic colonoscopy.

## Conclusions

Despite relatively low individual colonoscopy volumes, cecal intubation rates are meeting the recommendations suggested by the adult guidelines. TI intubation rates were lower, and there was a high degree of variability, but it was still over the 80% level as a group. Indications for pediatric colonoscopy usually include the need for TI intubation; this should be a competency objective in addition to the cecal intubation. This is contradictory to the current adult colonoscopy guidelines.
